# Facile Synthesis of chitosan-g-PVP/f-MWCNTs for application in Cu(II) ions removal and for bacterial growth inhibition in aqueous solutions

**DOI:** 10.1038/s41598-022-22332-8

**Published:** 2022-10-17

**Authors:** Samira T. Rabie, Yasser Mahmoud A. Mohamed, Reham A. Abdel-Monem, Hossam A. El Nazer

**Affiliations:** Photochemistry Department, National Research Center, Dokki, Giza, 12622 Egypt

**Keywords:** Chemistry, Materials science, Nanoscience and technology

## Abstract

Herein in this study, chitosan-grafted-4-vinylpyridine (Cs-g-PVP) and two polymeric hybrids of Cs-g-PVP/f-MWCNTs (I and II) with 3wt% and 5wt% f-MWCNTs, respectively were prepared, characterized and used as adsorbent for the removal of Cu(II) ions from aqueous solutions in a batch process The obtained Cs-g-PVP was characterized using Fourier transform infrared spectroscopy (FT-IR) to identify its surface functional groups, in addition thermal gravimetric analysis (TGA) and scanning electron microscopy (SEM) were performed to assess the thermal stability, the morphology and the elemental analysis of the obtained Cs-g-PVP and Cs-g-PVP/f-MWCNTs (I and II). Energy dispersive X-ray (EDX) with mapping analysis was obtained for Cs-g-PVP/Cu and Cs-g-PVP/f-MWCNTs/Cu samples that was confirming on the performance of adsorption batch process. The applicability of Langmuir adsorption isotherms was evaluated to better understand the adsorption process. Additionally, antibacterial activity of the Cs-g-PVP and the two polymeric hybrids Cs-g-PVP/f-MWCNTs (I and II) was evaluated against three Gram + ve bacteria (*Staphylococcus aurous*, *Bacillus Subtitles* and *Streptococcus faecalis*) and three Gram –ve bacteria (*Escherichia coli*, *Pseudomonas aeruginosa* and *Neisseria gonorrhoeae*. The results showed that the efficiency of Cs-g-PVP copolymer was improved after inclusion of the f-MWCNTs substrate towards adsorption of Cu(II) ions and antibacterial activity as well.

## Introduction

Chitosan (Cs) is a natural biopolymer with a linear polysaccharide structure and is found as an N-deacetylated chitin derivative^1^. Chitosan possesses a number of desirable characteristics, including biocompatibility, biodegradability, antibacterial activity, non-toxicity, and a variety of other physical features^2^. In addition, the presence of both amine and hydroxyl groups as chelating sites for metal ions, chitosan can be regarded as a suitable chelating agent^3–5^. Graft copolymerization of chitosan is a significant modification approach for affording new and various molecular designs of chitosan-based copolymers^6–8^. It was investigated that the chemical grafting of chitosan can improve some characteristics such as chelating affinity^9,10^, antibacterial activity^11^ and other features for a variety of applications including medicine, cosmetics, food processing, wastewater treatment, and environmental protection^12–14^. Recently, several studies reported the utility of grafted Cs as unconventional adsorbent for removal metallic contaminants that showed high metal adsorption affinities^15–17^. In continuation to our previous work for developing of new materials for wastewater treatment^18–20^, herein in this study we aimed to investigate the modification of chitosan by its reaction with 4-vinylpyridine (4-VP) to produce Cs-g-PVP copolymer and then in another series of experiments, functionalized multiwall carbon nanotubes (f-MWCNTs) were added to the prepared copolymer to obtain Cs-g-PVP/f-MWCNTs with the aim of improving the physisorption properties. To the best of our knowledge, few protocols reported the use homopolymer of PVP^21^ or copolymers containing 4-vinylpyridine in the removal of metal ions removal^22–25^. In addition, the use of MWCNTs as inorganic fillers in polymeric substrate reinforcement to be exhibited additional advantages and potential features had been studied previously^26^. As an example, alumina and functionalized MWCNTs were incorporated into chitosan to increase certain of its physicochemical qualities, such as thermal stability, while reducing the chitosan's swell ability and solubility. Also, it was investigated that Cs-alumina/f-MWCNTs nanocomposites have an antibacterial effect comparable to pure chitosan^27^. In another study, upon the treatment of MWCNTs with 8-hydroxyquinoline the formed composite was used to remove Cu (II), Cd (II), Pb (II), and Zn (II) from aqueous solutions^28^. Hence, we focused in this work on the design and characterization of a chitosan grafted-4-vinylpyridine copolymer to be applied as adsorbent for Cu(II) ions removal from aqueous solution and as antibacterial agent. In addition, the aim of this study was extended to prepare two new hybrids based on f-MWCNTs-based Cs-g-PVP copolymer to investigate their impact on Cu(II) ions removal in aqueous solution and bacterial growth suppression.

## Experimental

### Materials

Chitosan (MW 100–300 kDa, 82% degree of deacetylation) was purchased from Across Organics, Belgium. Potassium persulfate (KPS), CuCl_2_.2H_2_O, 4-vinylpyridine and MWCNTs were supplied from Sigma Aldrich Company. All other fine chemicals were of fine grades and all solvents were distilled before use.

### Characterization techniques and analysis

#### Fourier transform infrared (FTIR) spectroscopy

FTIR spectra were recorded on Shimadzu IR-Spectrometer (FTIR 8201) Japan, at room temperature within the wavenumber range of 4000–400 cm^−1^ using KBr discs.

#### Thermogravimetric analysis (TGA)

Thermogravimetric analysis was carried out on TGA-50H thermogravimetric analyzer, Shimadzu, Japan. Samples were heated up to 800 °C in a platinum pan with a heating rate of 10 °C/min, in N_2_ atmosphere of flow rate 25 mL/min.

#### Scanning electronic microscopy (SEM)

The dry samples were spread on a conducting adhesive tape, pasted on a metallic stub. The morphologies of the tested samples were investigated and imaged with scanning electron microscope (SEM) (QUANTA FEG 250 ESEM, USA). This was accompanied by energy dispersive X-ray spectroscopy (EDAX AMETEK Inc.; Mahwah, NJ, USA) at an acceleration voltage of 15 kV. The films were fixed on the surface of a sticky tape.

### Preparation of chitosan graft-poly(4-vinylpyridine)

**Figure 1:**
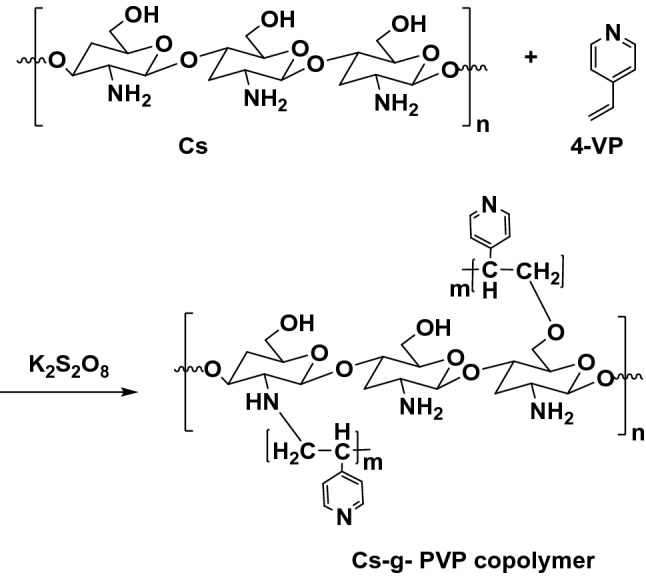
Preparation of graft copolymerization of chitosan and 4-vinylpyridine monomers.

Chitosan solution was prepared by dissolving 0.8 g of chitosan in 100 ml of 1% aqueous acetic acid solution and was placed in a flat bottomed three necked flask with stirring and the reaction temperature was raised to 60 °C. Then, nitrogen gas was purged into the reaction mixture at a constant temperature (ca ~ 60 °C) with continuous stirring. Freshly prepared potassium persulfate solution (3 × 10^–2^ mol/L) was added and then (2 mol/L) of 4-vinylpyridine was subsequently added by dropwise addition. The reaction was conducted for 2 h with stirring and continued for another 15 min at room temperature. Then the reaction product was precipitated out with 10% of (NaOH/MeOH) mixture, filtered, and dried. It was subjected to Soxhlet extraction for 8–12 h using N,N-dimethylformamide (DMF) to solubilize and remove any homopolymer^29^. The preparation of the chitosan grafted copolymer was illustrated in Fig. 1.

The graft yield (G%), the grafting efficiency (GE%) and the amount of homopolymer (H%) formed were calculated according to the following equation

1 $$ {\text{Graft yield }}\left( {{\text{G\% }}} \right) \, = \, \left[ {\left( {{\text{W}}_{1} - {\text{W}}_{0} } \right)/{\text{W}}_{0} } \right] \, \times \, 100 $$

2 $$ {\text{Grafting efficiency }}({\text{GE}}\% ) \, = \, \left( {{\text{W}}_{{1}} /{\text{W}}_{{2}} } \right) \, \times { 1}00 $$

where W_0_, W_1_ are the weights of initial matrix and grafted matrix, respectively^30^.

### Preparation of functionalized and Cs-g-PVP/f-MWCNTs hybrids

**Figure 2:**
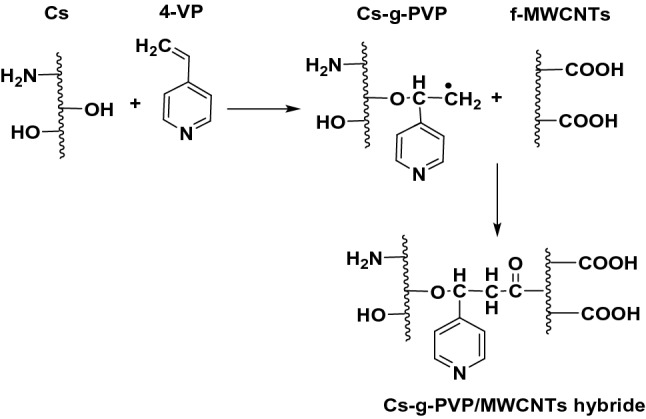
Formation of Cs-g-PVP/f-MWCNT hybrids (I or II) with different proportions of MWCNTs (3w% or 5w%) in regards to Cs-g-PVP.

Functionalization of MWCNTs (f-MWCNTs) was performed as reported before^31^. MWCNTs (0.5 g) were dispersed in 100 mL 65% nitric acid at 120 °C for 15 h under magnetic stirring. The obtained solution was then washed with water several times to neutrality, filtered and finally dried at 80 °C for 12 h to obtain the f-MWCNTs sample. Then, to a predetermined weight of the grafted copolymer Cs-g-PVP (1 g) in 20 mL of distilled water, f-MWCNTs (30 mg, 3wt%) or (50 mg, 5wt%) by weight of the grafted copolymer was added and left for 30 min under magnetic stirring and then sonicated for another 30 min to obtain Cs-g-PVC/f-MWCNTs hybrids. A schematic representation was given to explain the formation of Cs-g-PVP/f-MWCNT hybrid, Fig. 2.

### Cu(II) ions adsorption measurements using a batch method

The batch adsorption experiments were performed by taking 20 mL of CuCl_2_.2H_2_O solution with a concentration of 5 × 10^–3^ mol/L in the Erlenmeyer flask to afford a solution with pH 7. The other two solutions of CuCl_2_ with pH 4 and pH 9 were obtained by adjusting pH level using acetic acid/sodium acetate (AcOH/NaOAc) and NH_4_OH/NH_4_Cl buffer solutions to study the metal ions uptake at both the acidic and alkaline the mediums, respectively. The used adsorbent mass (0.05 g, 0.10 g, 0.15 g and 0.20 g) as equivalent to concentrations (2.5 g/L, 5 g/L, 7.5 g/L and 10 g/L) were added until and the adsorption equilibrium was reached after 60 min at 25 °C. After separation of the phases by centrifugation (10,000 rpm), the amount of Cu(II) ions uptake of the.

The removal efficiency (R%) was calculated using the formula (Eq. 3):

3 $$ {\text{R }}\% = \, \left( {{\text{C}}_{0} - {\text{C}}_{e} /{\text{C}}_{0} } \right) \times 100 $$

C_0_ = Initial concentration of Cu(II) ions in the solution. C_e_: The equilibrium concentration of Cu(II) ions.

### Antibacterial activity

Antibacterial activity of the tested samples was evaluated using a modified Kirby-Bauer disc diffusion method^32^. Briefly, 100 mL of the bacteria were grown in 10 mL of fresh media until they reached a count of approximately 108 cells/mL for bacteria^33^. 100 mL of microbial suspension were spread onto agar plates corresponding to the broth in which they were maintained. Isolated colonies of each organism, that might be playing a pathogenic role, should be selected from primary agar plates. They were examined for susceptibility by disc diffusion method^34,35^. Of the many media available, National Committee of Clinical Laboratory Standards (NCCLS) recommended Mueller–Hinton agar due to its good results in batch to-batch reproducibility. Antibacterial activity of the prepared hydrogels was investigated against three types of Gram + ve bacteria (*S. aurous*, *B. Subtitles* and *S. faecalis*) and three Gram –ve bacteria (*E. coli*, *P. aeruginosa* and *N. gonorrhoeae*. Plates are inoculated with bacteria at 35–37 °C for 24–48 h^32^. Standard discs of Ampicillin (Antibacterial agent) have served as positive controls for antibacterial activity but filter discs impregnated with 10 mL of solvent (distilled water, chloroform, DMSO) have been used as a negative control. The agar used is Meuller-Hinton agar that is rigorously tested for composition and pH. Further, the depth of the agar in the plate is considered to be a factor in the disc diffusion method. This method is well documented and standard inhibition zones have been determined for susceptible and resistant values. Blank paper discs (Schleicher and Schuell, Spain) with a diameter of 8.0 mm were impregnated with 10 m of tested concentration of the stock solutions. When a filter paper disc, impregnated with a tested chemical- is placed on agar, the chemical will diffuse from the disc into the agar. This diffusion will place the chemical on the agar only around the disc. The size of the area of chemical in filtration around the disc was determined by solubility of the chemical and its molecular size. If an organism is placed on the agar, it will not grow around the disc if it is susceptible to the chemical. This area of no growth around the disc is known as a “zone of Inhibition” or “clear zone” for the disc diffusion, the zone diameters were measured with slipping calipers of the NCCLS^33^. Agar based methods such as E-test and disc diffusion are considered to be good alternatives because they are simpler and faster than broth-based methods^36,37^.

## Results and discussion

In a first series of experiments, chitosan-grafted-poly(4-vinylpyridine) (Cs-g-PVP) copolymer was synthesized by grafting of 4-vinylpyridine (4-VP) onto chitosan in presence of a potassium persulfate through the heterogeneous radical polymerization as described in the experimental section. The grafting (G %) and grafting efficiencies (GE%) were calculated to be 92% and 236%, respectively. The prepared copolymer was characterized by using Fourier transformation infrared spectroscopic analysis to confirm the grafted copolymer that had been formed. In a second series of experiments, Cs-g-PVP/f-MWCNTs hybrids containing 3wt% (hybrid I) and 5wt% (hybrid II) of f-MWCNTs were also prepared under ultrasound irradiation conditions. In a third series of experiments, the application of the as-prepared grafted copolymer and its hybrids with f-MWCNTs (I and II) toward Cu(II) ions uptake was investigated using batch adsorption method. Characterizations of Cs-g-PVP and the Cs-g-PVP/f-MWCNTs hybrids (I and II) were determined via thermal gravimetric analysis (TGA), scanning electron microscopy (SEM). Different affecting parameters on the adsorption process were studied such as pH and the adsorbent mass. In addition, antimicrobial activity of the all-prepared samples was evaluated. The efficiency of the Cu(II) ions uptake was determined by % metal removal was studied.

### FTIR analysis

**Figure 3:**
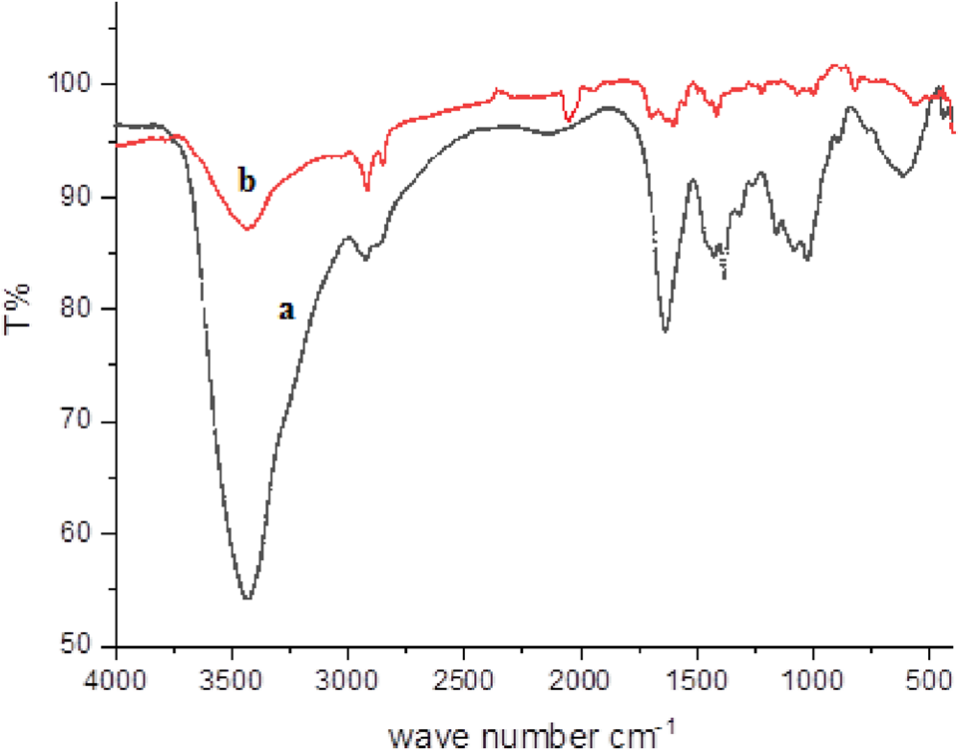
IR spectra of (**a**) Cs and (**b**) Cs-g-PVP.

The evidence of the formation of Cs-g-PVP was achieved by FTIR analysis. Figure 3a,b represented the FTIR spectrum of both Cs and Cs-g-PVP. It was clearly shown a strong IR band at 3433 cm^−1^ which should be assigned to the symmetrical stretching vibration of the OH groups, the extension vibration of the NH_2_, and the intermolecular hydrogen bonds of the polysaccharide. There are also two IR peaks appeared at 2918 and 2851 cm^−1^ that may be due to the aliphatic CH_2_ and CH. The C–O absorption peak of the hydroxyl group was observed to 1068 cm^−1^. In Fig. 1, the grafting of 4-vinylpyridine onto chitosan was confirmed by the presence of C = N groups band at about 2120 cm^−1^ from the poly 4-vinylpyridine chains and the –CH bending of the aromatic ring of the vinyl pyridine that appeared around 746, 821, and 1601 cm^−1^ and this is very indicative to the grafting copolymerization of vinyl pyridine moiety with chitosan^38^.

### Thermogravimetric analysis (TGA)

**Figure 4:**
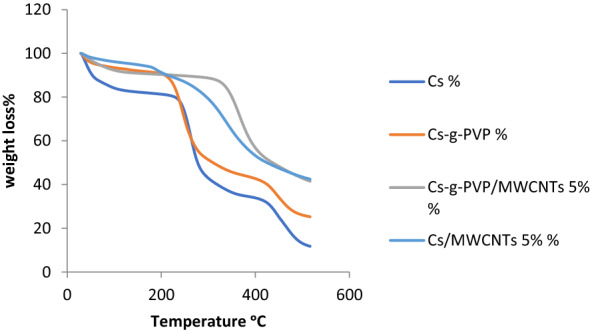
Thermogram of Cs, Cs-g-PVP, Cs-g-PVP/f-MWCNTs(5wt%) and Cs/f-MWCNTs(5wt%).

This analysis measures the thermal stability of chitosan and the prepared polymeric compound Cs-g-PVP. In addition, the effect of adding f-MWCNTs with amount 5wt% to both Cs and the Cs-g-PVP to yield Cs/f-MWCNTs and Cs-g-PVP/f-MWCNTs hybrids was investigated. The results revealed that both Cs/f-MWCNTs and Cs-g-PVP/f-MWCNTs hybrids showed significant higher thermal stability than either Cs or Cs-g-PVP (Fig. 4). The thermogram revealed that Cs-g-PVP has a relative higher thermal stability than Cs in terms of both initial decomposition temperature (IDT) and residual weight below 500 °C. For example, at 250 °C, the recorded weight loss of Cs, Cs-g-PVP, Cs/MWCNTs and Cs-g-PVP/MWCNTs hybrids are 27%, 31%, 13% and 10%, respectively. The improved thermal stability of the two hybrids was observed at the decomposition temperature of 300 °C, since the weight loss of Cs/f-MWCNTs and Cs-g-PVP/f-MWCNTs hybrids are 21% and 11%, whereas the percentages weight loss of both Cs and Cs-g-PVP are 57% and 49%, respectively. The observed weight loss in the early stages of degradation for all investigated samples might be attributed to any attached water residues or volatiles being released from the polymeric chains. The observed degradation of Cs could be due to dehydration and depolymerization of Cs units, which increases as the polymeric network structure proceeds^39,40^. However, the presence of a PVP block in Cs-g-PVP grafted copolymer improved the thermal stability due to the presence of the aromatic ring in the chemical structure of PVP. It was recorded that the weight loss of Cs and Cs-g-PVP hybrids at 400 °C was 66% and 57%, respectively, while Cs/f-MWCNTs and Cs-g-PVP/f-MWCNT hybrids lost 47% and 43% of their weight at the same temperature. At 515 °C, both Cs/f-MWCNTs and Cs-g-PVP/f-MWCNTs hybrids have almost the same weight loss of about 58%, whereas Cs and Cs-g-PVP exhibited weight loss of 88% and 75%, respectively. The thermal properties of the samples under investigation were represented in Table 1 that illustrate the temperatures at which they lost 10% and 50% of their weight and the residual weights of the tested samples at 400 °C and 500 °C.

**Table 1:** Thermal properties of various investigated samples.

Sample	*T_10%_	T_50%_	**R_400_°C	R_500_°C
Cs	53	277	34	13
Cs-g-PVP	207	310	43	26
Cs/f-MWCNTs (5wt%)	213	423	53	43
Cs-g-PVP/f-MWCNTs (5wt%)	229	436	57	43

*T = The temperatures at which the material lost 10% and 50% of their weight, **R = The residual weights of the tested samples at 400 °C and 500 °C.

From the results, it was demonstrated that the thermal stability of Cs and the grafted copolymeric matrix of Cs-g-PVP was improved by incorporation f-MWCNTs. It could be referred to the barrier function of the f-MWCNTs that might be responsible for the improvement in thermal stability.

### Scanning electron microscopy

**Figure 5:**
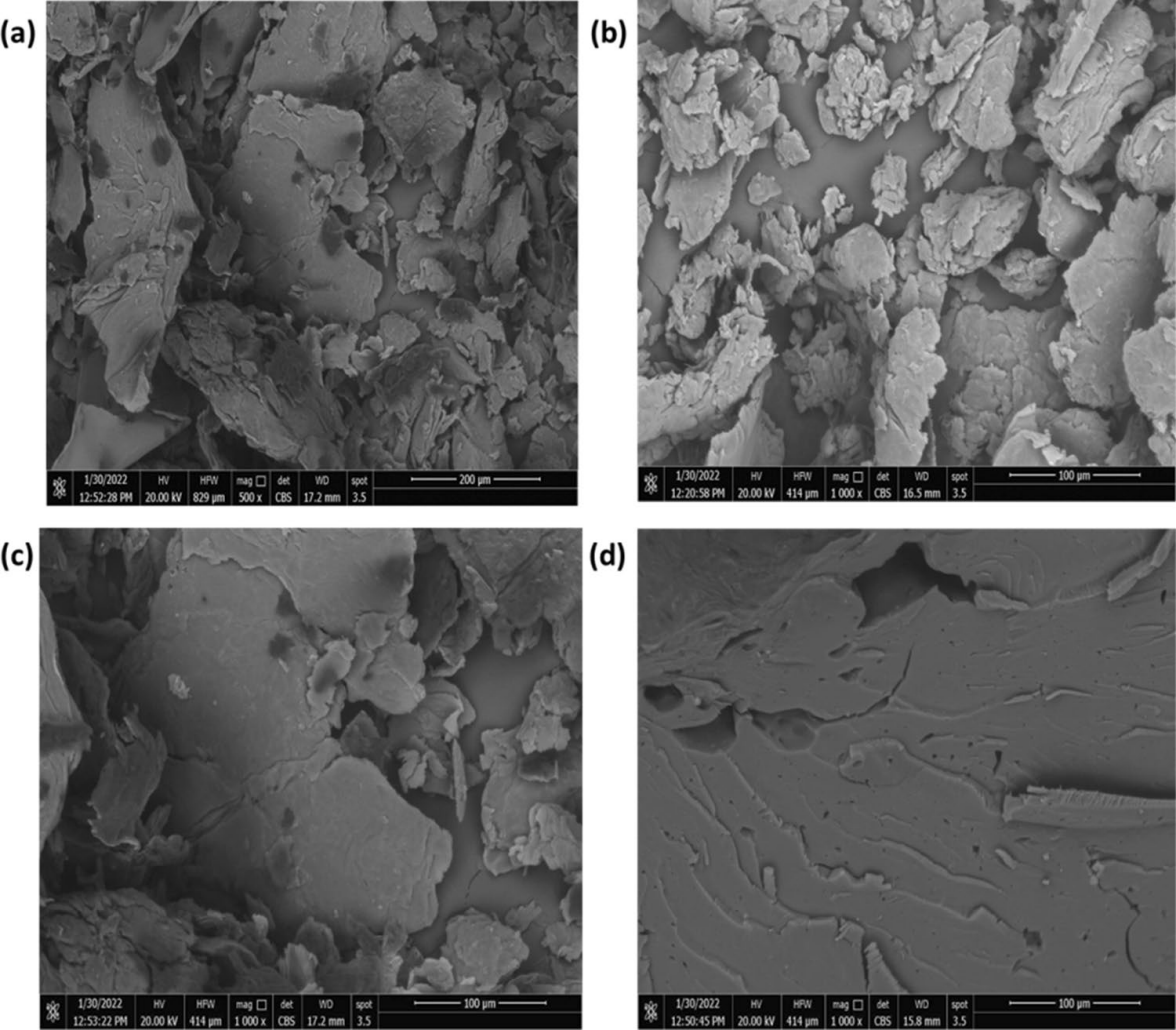
SEM images of Cs (**a**), Cs-g-PVP (**b**), Cs-g-PVP/f-MWCNT 3wt% (**c**), and Cs-g-PVP/f-MWCNT 5wt% (**d**).

SEM images of Cs and Cs-g-PVP Cs-g-PVP/f-MWCNTs (I and II) hybrids and are shown in Fig. 5a–d. The morphology of the chitosan was depicted in Fig. 3a. Upon graphitization of Cs with 4-VP, to obtain Cs-g-PVP, an obvious change in the surface morphology of the grafted chitosan that gave a proof for conjugation of 4-VP blocks in the copolymer chains and this is represented in Fig. 5b. By treating Cs-g-PVP copolymer with f-MWCNTs (3wt% and 5wt%), it was apparently shown that the functionalized MWCNTs were bonded to chitosan-g-PVP. The f-MWCNTs were appeared in micron length sizes in SEM images (Fig. 5c,d) with distinct agglomeration in the surface of the formed polymeric composite.

### Batch adsorption studies

Batch adsorption experiments were performed using Cs-g-PVP, Cs-g-PVP/f-MWCNTs (I) and (II) hybrids (containing 3wt% and 5wt% by weight) as adsorbent. Herein, in this study the effect of composition of the polymeric materials, pH and adsorbent mass were investigated.

#### Adsorption of Cu(II) ions onto Cs-g-PVP copolymer under the effect of pH and adsorbent mass.

**Figure 6:**
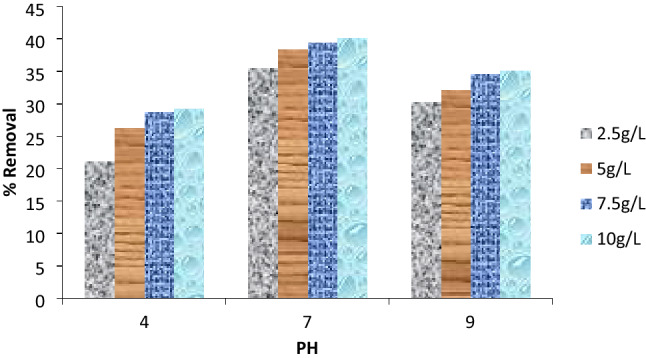
Cu(II) ions removal % as a function of pH and adsorbent masses.

The results of Cu(II) ions uptake by the produced Cs-g-PVP at different pH (4, 7 and 9) and with mass amount (2.5 g/L, 5 g/L, 7.5 g/L or 10 g/L) were shown in Fig. 6. It was revealed that when different masses of adsorbent were used at pH 4 and 7, the removal efficiency (R%) of metal ions was increased^41^, but there was an observable reduction in R% at pH 9. In a first set of experiments, when Cs-g-PVP was used with the mass amounts of 2.5 g/L, 5 g/L, 7.5 g/L and 10 g/L, the metal ions removal was detected as follows: 21.08%, 26.22%, 28.75%, and 29.17% at pH 4. However, at pH 7, the percentages of metal ions removal for the same copolymer masses are 35.54%, 38.38%, 39.32%, and 40.09%. However, at pH 9 these percentages, the metal ions removal efficiency were reduced as follows: 30.27%, 32.17%, 34.59% and 35.12%. The increase of metal removal by the increase of pH from 4 to 7 may be due to the remarkable hydrolysis of the copper (II) ions at higher pH and this may compete with polymer chelation process. In all cases, the pH highly affects the oxygen atoms of OH or the lone pairs of electrons on the nitrogen atoms of the adsorbent. Hence it was indicated that both the pH and the adsorbent mass have an impact on the metal ions removal of copper (II) from aqueous solutions.

#### The activity of Cs, Cs/ f-MWCNTs (I or II), Cs-g-PVP/f-MWCNTs (I or II) hybrids towards Cu(II) ions removal

**Figure 7:**
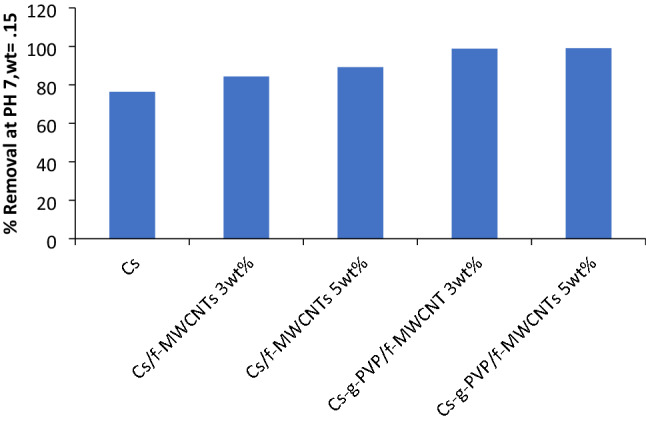
Removal efficiency % by Cs, Cs/f-MWCNTs hybrids containing 3wt% and 5wt% by weight of f-MWCNTs and Cs-g-PVP/f-MWCNTs hybrids containing 3wt% and 5wt% by weight of f-MWCNTs using 0.15 g adsorbent at pH 7.

The performance of Cs and Cs/f-MWCNTs hybrids (I or II), containing 3wt% and 5wt% of f-MWCNTs towards the removal of Cu(II) ions was investigated. Adsorbent mass was taken as fixed amount 10 g/L at pH 7. The obtained removal efficiency for the removal of Cu (II) ions were 76.38%, 84.36% and 89.19%, respectively for the three tested samples. The observable enhancement of metal ions removal % using the aforementioned compound was confirming on the important role of f-MWCNTs. This may be due to the presence of more negatively charged oxygen-containing groups on the oxidized or functionalized form of MWCNTs^31^ that have the affinity to attract the Cu(II) ions from the aqueous solutions. In another set of experiments, the adsorption capacity of Cs-g-PVP/f-MWCNTs (hybrid I) or (hybrid II) (10 g/L at pH 7) was evaluated. It was determined that the removal efficiencies of Cu(II) ions uptake were 98.80% or 99.01%, respectively (Fig. 7).

**Figure 8:**
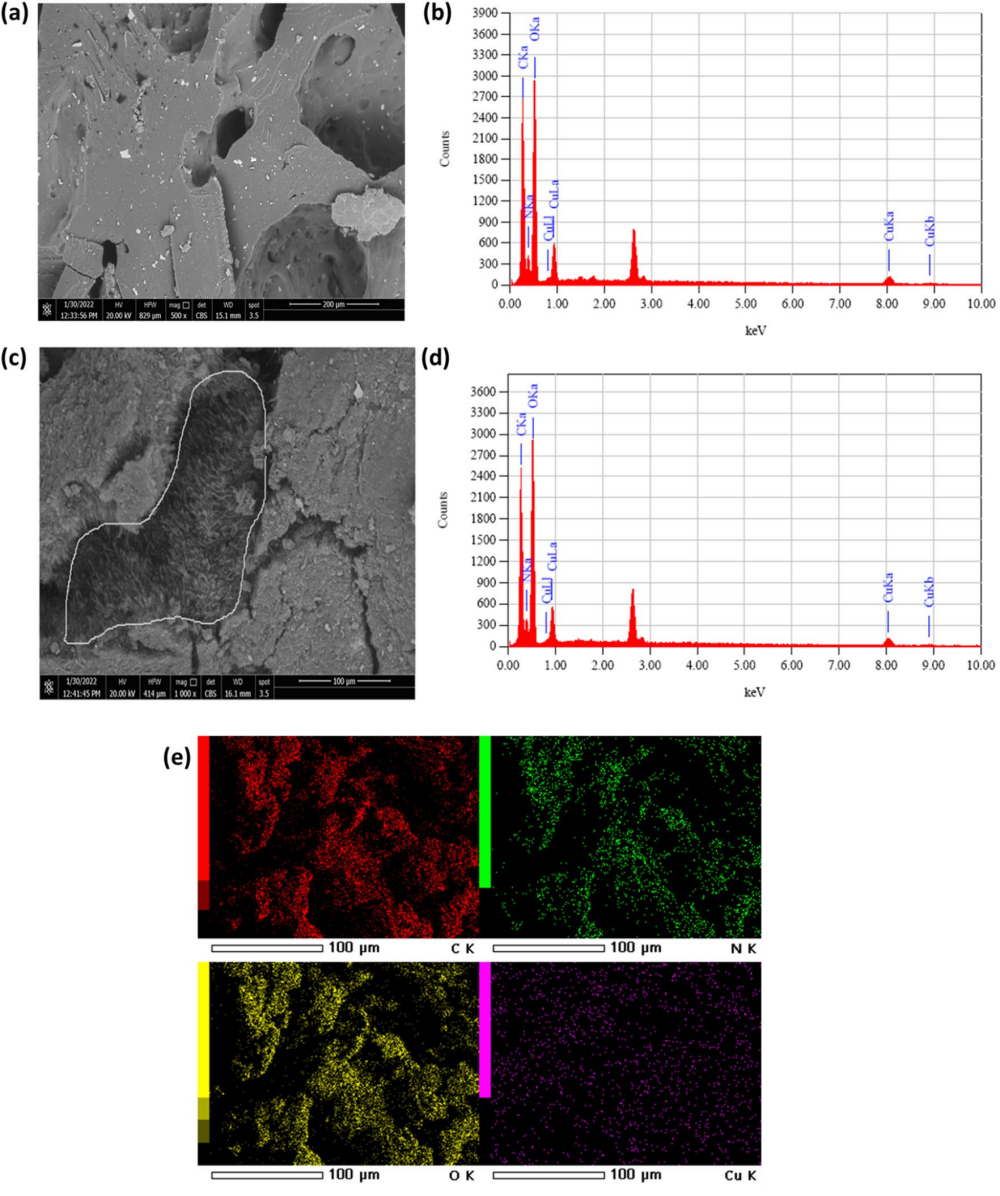
(**a**) SEM image of Cs-g-PVP/CuCl_2_ (**b**) EDX profile of Cs-g-PVP/CuCl_2_; (**c**) SEM of Cs-g-PVP/MWCNTs/CuCl_2_; (**d**) EDX profile of Cs-g-PVP/f-MWCNTs/CuCl_2_ and (**e**) EDX mapping analysis of elemental constitutes of Cs-g-PVC/f-MWCNTs/CuCl_2_ hybrid.

As an evidence for the incorporation of Cu(II) ions in Cs-g-PVP and Cs-g-PVP/f-MWCNTs, SEM analyses were performed (Fig. 8a,c). In Fig. 8a, it was shown that the presence of copper metal ions on the Cs-g-PVP polymeric surface that appeared as bright spots. In EDX pattern, the chemical compositions of Cs-g-PVP/Cu in weight% showed (C, N, O and Cu) was found (57.2 wt%, 5.4 wt%, 25.4 w% and 12.0 wt%) (Fig. 8b). The SEM image of the as-prepared Cs-g-PVP/f-MWCNTs 5wt% (hybrid II) after Cu(II) ions adsorption process (Fig. 8c) showed that f-MWCNTs appeared as sequestered within regions of Cs-g-PVP that probably considered as the more hydrophobic phase on the polymeric hybrid. The chemical compositions of Cs-g-PVP/f-MWCNTs/Cu showed (C, N, O and Cu) was estimated to be (56.1 wt%, 5.2 wt%, 26.5 w% and 12.1 wt%) (Fig. 8d). Also mapping of each element in Cs-g-PVP/f-MWCNTs/Cu sample was represented in Fig. 8e. From these results, it was confirmed that as prepared Cs-g-PVP and Cs-g-PVP/f-MWCNTs were efficient materials for Cu(II) adsorption in aqueous solutions.

#### Adsorption isotherm study

The equilibrium capacity to adsorb Cu(II) ions for Cs-g-PVP-f-MWCNTs (hybrid II) was described Langmuir isotherm, describing the ratio of adsorbed Cu(II) ions and that remained in solution after equilibrium^42^. In this study, Cu(II) ions adsorption was measured at optimum conditions, at 25 °C. The Langmuir equation was used to calculate the maximum adsorption capacity, with complete monolayer coverage of Cs-g-PVP/f-MWCNTs (hybrid II) surfaces. This mode was expressed by Eq. (4).

4 $$ {\text{1/q}}_{{\text{e}}} {\text{ = 1/q}}_{{{\text{max}}}} {\text{ + 1/q}}_{{{\text{max}}}} {\text{bC}}_{{\text{e}}} $$

q_e_ and q_max_: The equilibrium and maximum adsorption capacity of Cu(II) ions (mg/g), respectively. C_e_: the equilibrium concentration of adsorbent (mg/L). b: and Langmuir constant (L/mg).

**Figure 9:**
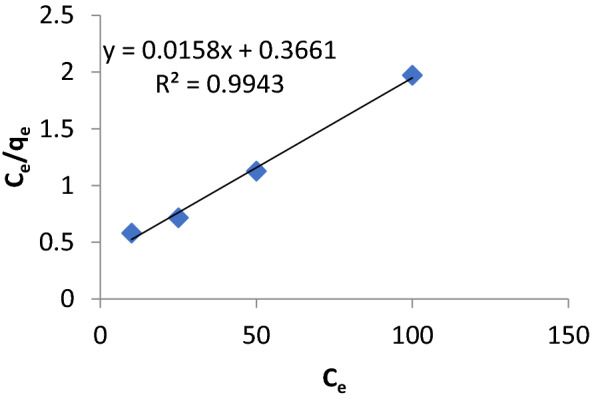
Langmuir isotherm model for Cu(II) ions adsorption onto Cs-g-PVP/f-MWCNTs (hybrid II).

In Fig. 9, it was obvious that the adsorption of Cu(II) ions was fitted with Langmuir model. The fitting parameters were represented in Table 2, with correlation coefficient (R^2^ = 0.9943).

**Table 2:** Adsorption isotherm parameters for Cu(II) ions adsorption onto Cs-g-PVP/f-MWCNTs (hybrid II) surface adsorbent.

Parameters	Values
q_max_	63.29 mg/g
b	0.043 L/mg
R^2^	0.9943

As seen from the above mentioned results, the maximum adsorption capacity (q_max_) was 63.29 mg/g. It can be assumed Cu(II) ions was forming monolayer onto Cs-g-PVP/f-MWCNTs (hybrid II) surface.

#### The mechanism of Cu(II) adsorption onto Cs-g-PVP/f-MWCNTs (hybrid II) surface

**Figure 10:**
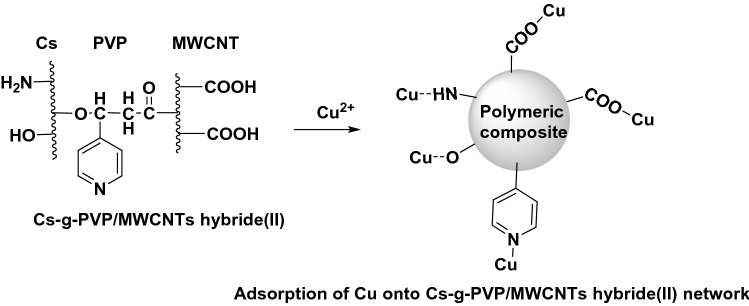
Postulated mechanism for Adsorption of Cu(II) onto Cs-g-PVP/f-MWCNT hybrids (II).

Figure 10 depicted a the adsorption behavior of Cu(II) onto the surface of the polymeric network. It can e observed that due to the presence of various polymer sites available for chelation such as NH_2_ and OH in chitosan part, pyridine in PVP part and COOH in MWCNT part facilitate the formation of ion-ion interaction between the adsorbent (polymeric hydride II) and adsorbate (Cu^2+^).

### Antibacterial activity

The antimicrobial activity of chitosan (Cs), chitosan grafted copolymer (Cs-g-PVP) and two grafted copolymers/f-MWCNTs hybrids (Cs-g-PVP/f-MWCNTs hybrids that containing 3wt% and 5wt% by weight of the MWCNTs) were evaluated against the gram + ve bacteria (*B*. *Subtitles*, *St*. *aurous* and *St*. *faecalis*) and the gram −ve bacterial strains (*E*. *coli*, *N*. *gonorrhoeae* and *P*. *aeruginosa*) that isolated from animal origin. Agar disk diffusion method was used for the determination of the preliminary antibacterial and Ampicillin was used as reference antibacterial drugs. All of the investigated samples showed, in vitro, antibacterial activity against the tested microorganisms. The results of antibacterial activity of the samples under investigation using inhibition zone method are listed in Table 3.

**Table 3:** The antimicrobial activities of the investigated samples against some G + ve and G -ve bacterial strains.

Sample	Inhibition zone diameter (mm/mg sample)					
	*Bacillus Subtitles*	*Staphylococcus aurous*	*Streptococcus faecalis*	*Escherichia coli*	*Neisseria gonorrhea*	*Pseudomonas gonorrhoeae*
	G + ve			G−ve		
DMSO	0.0	0.0	0.0	0.0	0.0	0.0
Ampicillin (100 µg/mL)	20	18	18	22	20	17
Cs	13	15	12	14	11	12
Cs-g- PVP	17	17	15	19	17	16
Cs-g-PVP/f- MWCNTs (3wt%)	19	19	17	25	22	18
Cs-g-PVP/f-MWCNTs (5wt%)	20	21	17	26	24	21

The results showed that when compared to pristine chitosan, Cs-g-PVP had improved antibacterial activity against all strains of gram-positive and gram-negative bacteria. As a result, Cs has antibacterial efficacy against both types of bacterial strains in terms of inhibition zone diameter. The inhibition zone caused by Cs against the gram + ve bacteria (*B*. *Subtitles*, *St*. *aurous* and *St*. *faecalis*) are 13, 15 and 12 with efficiencies of 65%, 83% and 67%, respectively with respect to the reference drug. On the other hand, the antibacterial activity of by the effect of Cs against the gram −ve bacterial strains (*E*. *coli*, *N*. *gonorrhoeae* and *P*. *aeruginosa*) are 14, 11 and 12 with efficiencies of 64%, 55% and 71%, respectively when compared to the standard reference antibacterial drug. The insertion of 4-vinylpyridine (4-VP) as blocks via grafting copolymerization with Cs yielding Cs-g-PVP improved the antibacterial activity of this grafted copolymer against all tested microbes, according to the results of Table 3.

In comparison to the reference drug, the antibacterial efficiency for gram + ve bacteria (*B*. *Subtitles*, *St*. *aurous*, and *St*. *faecalis*) were 85, 94, and 83, respectively. The antibacterial activity of Cs-g-PVP against the gram −ve bacterial strains (*E*. *coli*, *N*. *gonorrhoeae* and *P*. *aeruginosa*) reached 86, 85 and 94%, respectively with respect to the reference antibacterial drug. As a result, the Cs-g-PVP exhibit better efficiency against G −ve bacterial strains than the G + ve kinds, according to the given results. These results could be explained by the electrostatic interaction between positively charged chitosan molecules and negatively charged microbial cell membranes, which is the first proposed accepted mechanism for chitosan antibacterial activity^43^.

The second predicted action mechanism is chitosan's binding to microbe DNA, which results in mRNA and protein synthesis suppression in the nuclei of bacteria due to chitosan penetration^44^. Based on the well-known biological properties of nitrogen containing six membered aromatic heterocyclic compounds and their derivatives^45^, the observed increased antibacterial activity of Cs-g-PVP can be attributed to the PVP blocks in the grafted copolymeric chains. Additionally, the hydrophobic character of 4-VP in the copolymer chains increases the density of positive charges on the system, which may favor microbial cell attachment to the antibacterial system. As a result, 4-VP-based polymers or copolymers are intriguing materials with a variety of uses, including antibacterial materials^46,47^. Despite its well-known antibacterial properties, applications of Cs are limited due to undesirable properties such as low solubility and chemical stability^48–50^. As a result, various modifications or the addition of reinforcement materials may give rise to improvement to the physicochemical properties of chitosan, allowing it to be used in a wider range of applications.

Multiwalled carbon nanotubes (MWCNTs) have thus been proposed as an excellent material to utilize as inorganic fillers for reinforcing or toughening polymeric materials^26^, in addition to the grafting of 4-VP onto chitosan performance. Two samples of Cs-g-PVP containing 3 and 5% by weight of MWCNTs were functionalized as stated in the experimental section, affording two hybrids of Cs-g-PVP/f-MWCNTs for testing their biological activity. Table 2 shows the antibacterial activity of the two hybrids against the bacterial strains that were examined. When compared to either chitosan or the reference drug, the results showed that the two hybrids had much higher antibacterial efficacy against both types of bacteria, G + ve and G −ve bacteria. Antibacterial activity of the hybrid containing 3% by weight of f-MWCNTs against G + ve bacterial strains (*B*. *Subtitles*, *St*. *aureus*, and *St*. *faecalis*) was 95%, 106%, and 94%, respectively, as compared to the conventional drug.

In comparison to the reference drug, the obtained antibacterial activity of the same sample against G −ve bacteria (*E. coli*, *N. gonorrhoeae*, and *P. aeruginosa*) was 114, 110, and 106 respectively. The antibacterial activity effectiveness of the other hybrid, which contained 5wt% by weight of f-MWCNTs, was the highest of the investigated samples. In comparison to the reference antibacterial drug, the inhibitory effect of this hybrid revealed inhibition zones of 20, 21, and 17 mm, respectively, with efficiency of 100, 117, and 94% against G + ve bacteria (*B*. *Subtitles*, *St*. *aurous*, and *St*. *faecalis*). When compared to the reference drug, the examined hybrid recorded inhibitory zones of 26, 24 and 21 mm for the G −ve bacterial strains (*E*. *coli*, *N*. *gonorrhoeae*, and *P*. *aeruginosa*), with efficiencies of 118, 120, and 124%, respectively. The presence of f-MWCNTs in the tested samples is responsible for the observed high antibacterial activity of the two hybrids. MWCNTs have been shown to have strong inhibitory effects on a variety of bacteria in previous work^51^.

All postulated mechanisms to explain the bactericidal effect of CNTs in general are not fully known, and there are numerous factors that influence its antibacterial action. The diameter, electrical structure, residual catalyst, length, surface functional group, and other parameters are among them^52^. In terms of the functionalized MWCNTs employed in this investigation, it has been noted that functionalization is a good way to improve their dispersion in different matrices while also increasing biocompatibility and lowering toxicity in human cells^53^. The optical density of *E. coli* was significantly reduced when amine or carboxyl moieties were added to MWCNT. According to certain observations, the functionalized carboxylated MWCNTs increased membrane roughness and hydrophilicity, which made them more resistant to bacterial adherence. These findings suggested that f-MWCNT surfaces could be used in the manufacture of medical devices and biomedical applications^54,55^.

**Figure 11:**
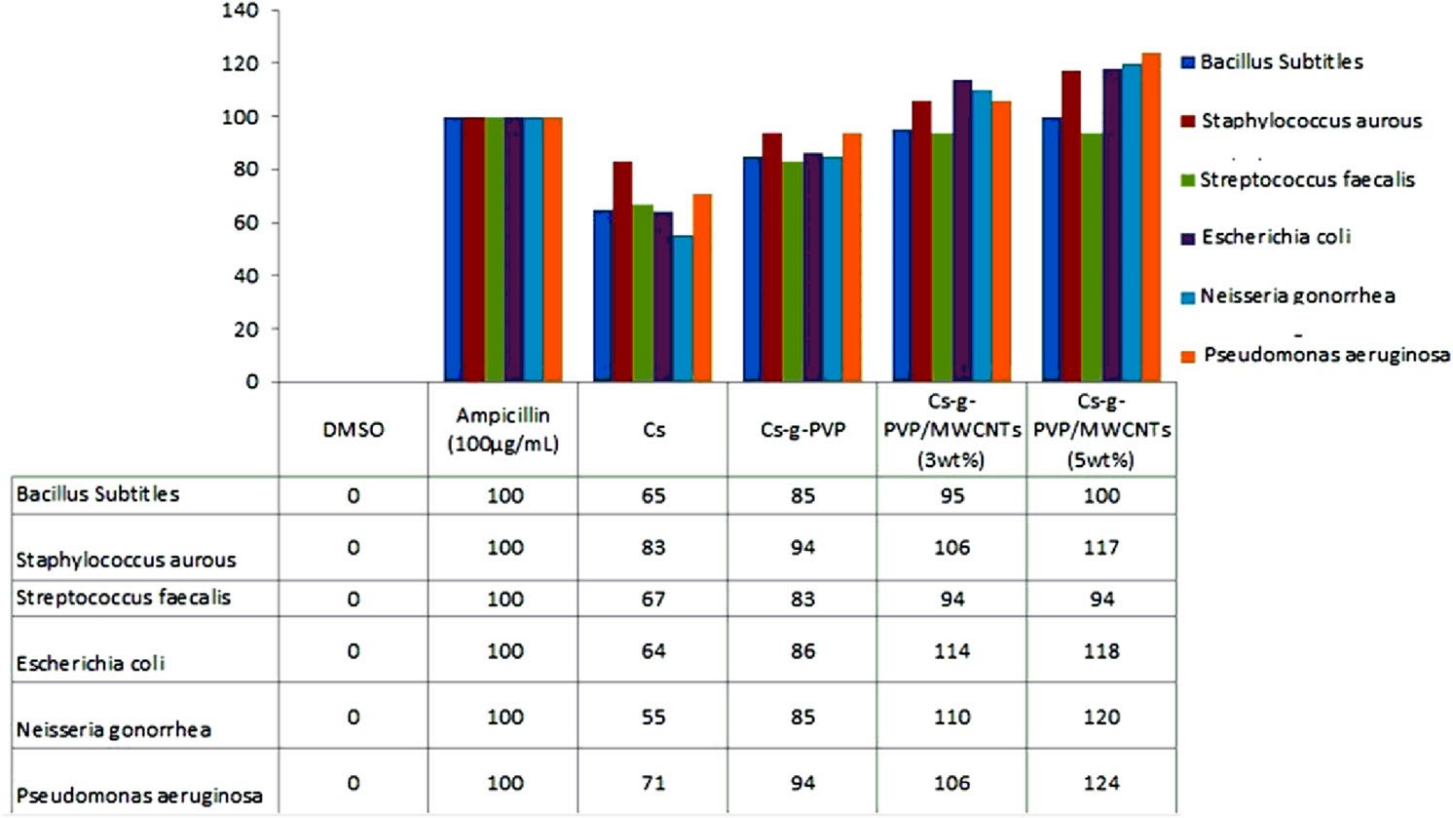
Antibacterial efficiency of the investigated samples with respect to the standard reference drug.

The incorporation of MWCNTs or f-MWCNTs into chitosan or other synthetic polymers to obtain potentially helpful antibacterial surfaces against Gram-positive and Gram-negative bacteria has been investigated. This could lead to the development of unique physicochemical properties for these polymeric substrates, as well as the prospect of using these composites in a variety of biological applications such as tissue engineering, bio-sensing, wound dressing, and drug administration^56,57^. In Fig. 11, it was illustrated the antibacterial efficiency of the investigated samples with respect to the standard reference drug.

## Practical applications and future research prospects

The design and development of highly efficient polymers towards the adsorption of heavy metal ions in wastewater has gained tremendous attention. An optimized procedure for batch adsorption process is demanded in which the reduction in the operational costs of the water treatment process could be investigated. In this protocol, the application of Cs-g-PVP/f-MWCNTs had offered practical solution to water pollution treatment. The presented technique was focused on the adsorption of Cu(II) ions as main pollutant in industrial wastewater. This report is beneficial for researchers or engineers who are working in environmental research or in related-water industry.

## Conclusions

In this study, it was investigated that the catalytic performance of Cs-g-PVP and Cs-g-PVP/f-MWCNTs hybrids was explored in the batch adsorption of Cu(II) ions in aqueous solutions as simulated wastewater. The results revealed that upon conjugation of f-MWCNTs with Cs-g-PVP, a covalent integration between f-MWCNTs and Cs-g-PVP have been accomplished. It was demonstrated that the addition of f-MWCNTs to Cs-g-PVP copolymer enhanced the thermal and chemical stability of the polymeric materials due to high tensile strengths, and are ultra-light weight of f-MWCNTs. The study was extended for testing the bioactivity of the as-prepared samples. It was revealed that Cs-g-PVP/f-MWCNTs (hybrid II, containing 5wt% f-MWCNTs), showed the highest antibacterial activity.

## Supplementary Information

Supplementary Information 1.

Supplementary Information 2.

Supplementary Information 3.

## Data Availability

All data generated or analyzed during this study are included in this published article [and its supplementary information files].
