# Supplementary material for: Facile Synthesis of chitosan-g-PVP/f-MWCNTs for application in Cu(II) ions removal and for bacterial growth inhibition in aqueous solutions
Source: Sci Rep. 2022 Oct 17;12:17354. doi: 10.1038/s41598-022-22332-8 (PMC9576794; doi:10.1038/s41598-022-22332-8)
Supplement: Supplementary file 1 — Supplementary Information 1. [file 41598_2022_22332_MOESM1_ESM.pdf]

Title : IMG1

Instrument : JCM-6000PLUS
Volt : 15.00 kV
Mag. : x 500
Date : 2022/01/18
Pixel : 512 x 384

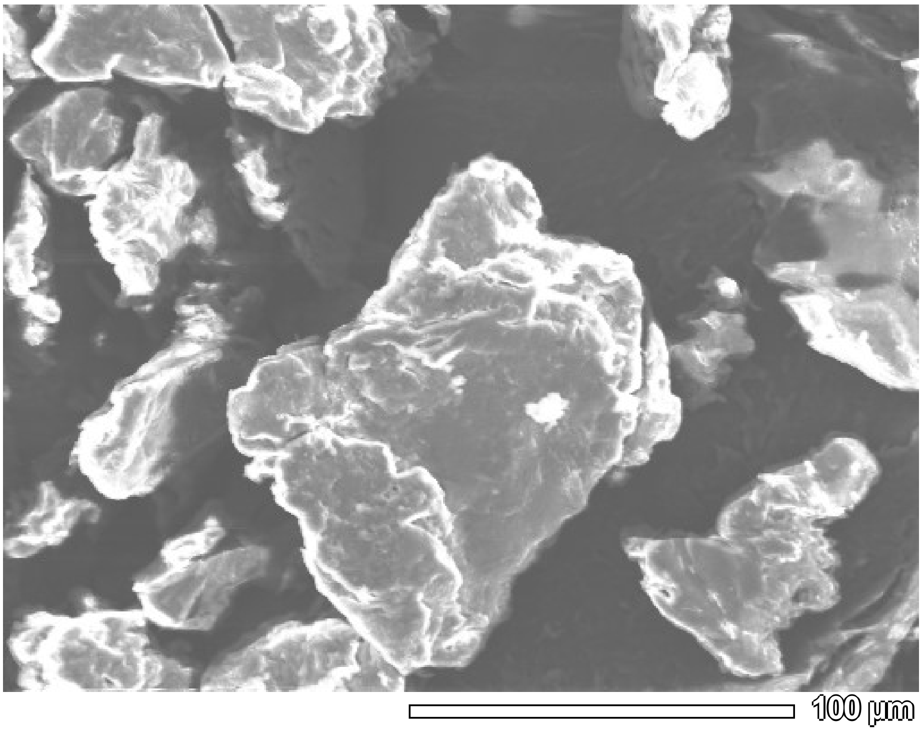

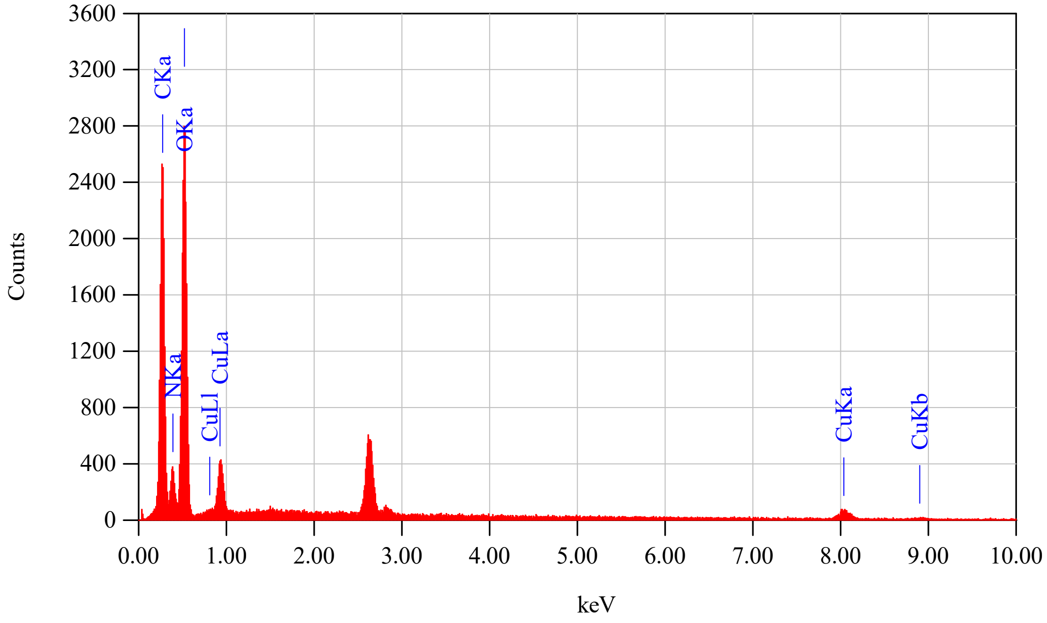

Acquisition Parameter
Instrument : JCM-6000PLUS
Acc. Voltage : 15.0 kV
Probe Current: 7.47500 nA
PHA mode : T3
Real Time : 50.77 sec
Live Time : 50.00 sec
Dead Time : 1 %
Counting Rate: 1495 cps
Energy Range : 0 - 20 keV

Thin Film Standardless Standardless Quantitative Analysis
Fitting Coefficient : 0.1550

Element	(keV)	Mass%	Counts	Sigma	Atom%	Compound	Mass%	Cation	K
C K	0.277	59.57	15319.88	0.31	69.40				2.5867
N K	0.392	5.53	2562.43	0.11	5.52				1.4347
O K (Ref.)	0.525	26.58	17680.36	0.23	23.24				1.0000
Cu K*	8.040	8.33	1007.06	0.41	1.83				5.5044
Total		100.00			100.00				
