# Supplementary material for: Facile Synthesis of chitosan-g-PVP/f-MWCNTs for application in Cu(II) ions removal and for bacterial growth inhibition in aqueous solutions
Source: Sci Rep. 2022 Oct 17;12:17354. doi: 10.1038/s41598-022-22332-8 (PMC9576794; doi:10.1038/s41598-022-22332-8)
Supplement: Supplementary file 2 — Supplementary Information 2. [file 41598_2022_22332_MOESM2_ESM.pdf]

View013

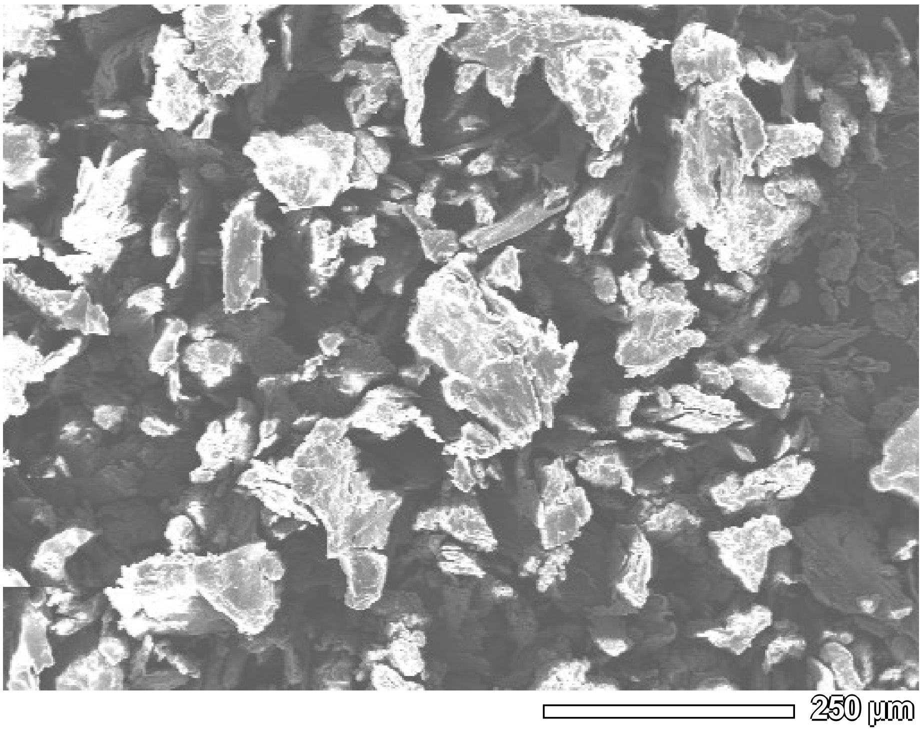

Title	:	IMG1
Instrument	:	JCM-6000PLUS
Volt	:	15.00 kV
Mag.	:	x 130
Date	:	2022/01/18
Pixel	:	512 x 384

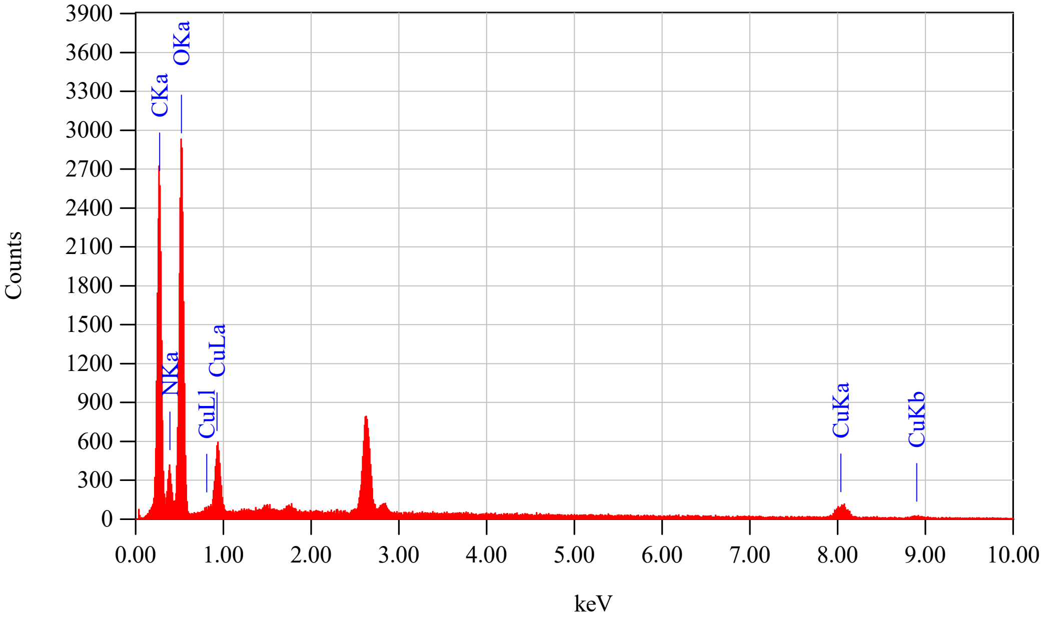

Acquisition Parameter

Instrument	:	JCM-6000PLUS
Acc. Voltage	:	15.0 kV
Probe Current	:	7.47500 nA
PHA mode	:	T3
Real Time	:	50.91 sec
Live Time	:	50.00 sec
Dead Time	:	1 %
Counting Rate	:	1716 cps
Energy Range	:	0 - 20 keV

Thin Film Standardless Standardless Quantitative Analysis
Fitting Coefficient : 0.1691

Element	(keV)	Mass%	Counts	Sigma	Atom%	Compound	Mass%	Cation	K
C K*	0.277	57.20	15972.18	0.29	68.78				2.5867
N K*	0.392	5.42	2727.31	0.10	5.59				1.4347
O K (Ref.)	0.525	25.38	18331.93	0.22	22.91				1.0000
Cu K*	8.040	12.00	1574.77	0.47	2.73				5.5044
Total		100.00			100.00				
