# Supplementary material for: Facile Synthesis of chitosan-g-PVP/f-MWCNTs for application in Cu(II) ions removal and for bacterial growth inhibition in aqueous solutions
Source: Sci Rep. 2022 Oct 17;12:17354. doi: 10.1038/s41598-022-22332-8 (PMC9576794; doi:10.1038/s41598-022-22332-8)
Supplement: Supplementary file 3 — Supplementary Information 3. [file 41598_2022_22332_MOESM3_ESM.pdf]

Supplementary Information

Facile Synthesis of chitosan-g-PVP/f-MWCNTs for application in Cu(II) ions removal and for bacterial growth inhibition in aqueous solutions

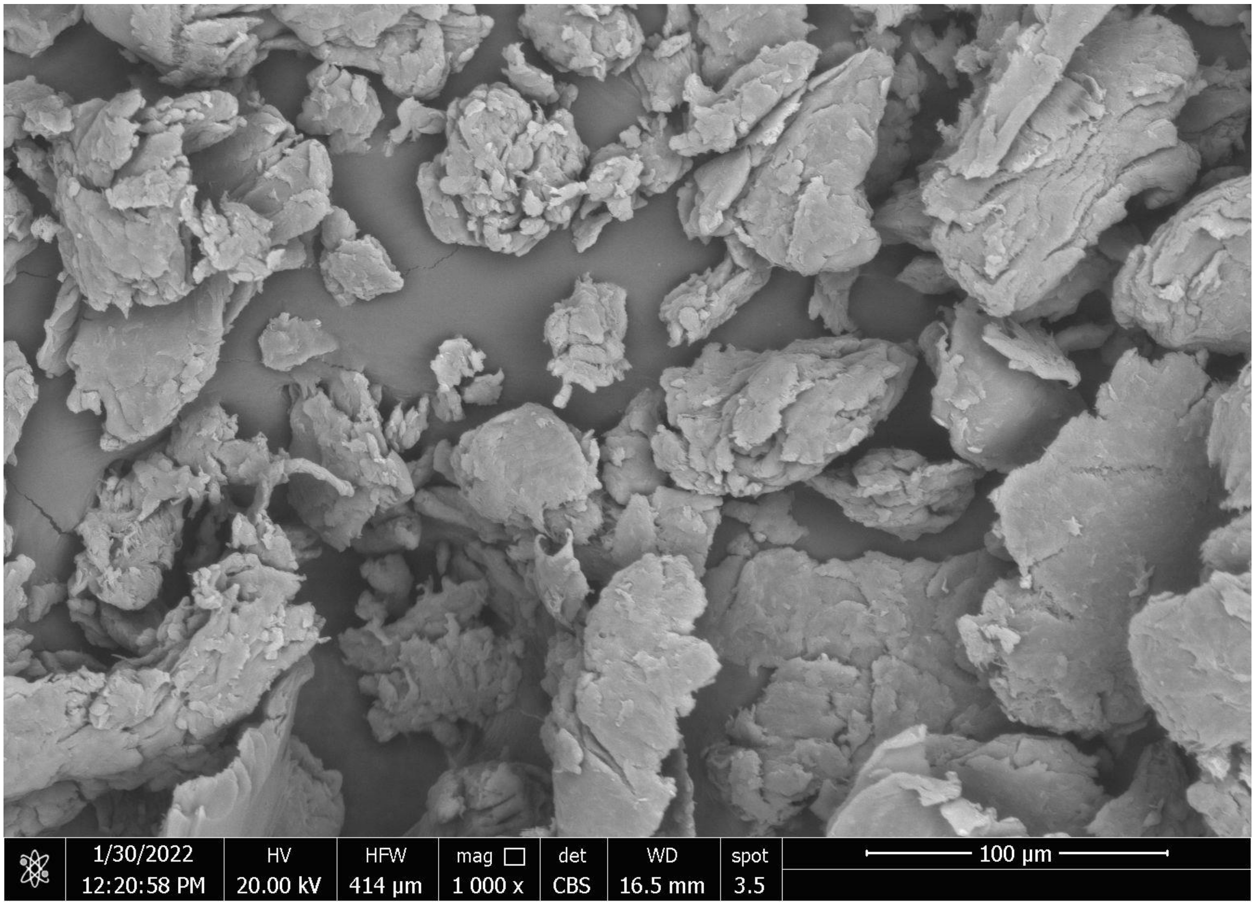

SEM image of Cs/$CuCl_2$

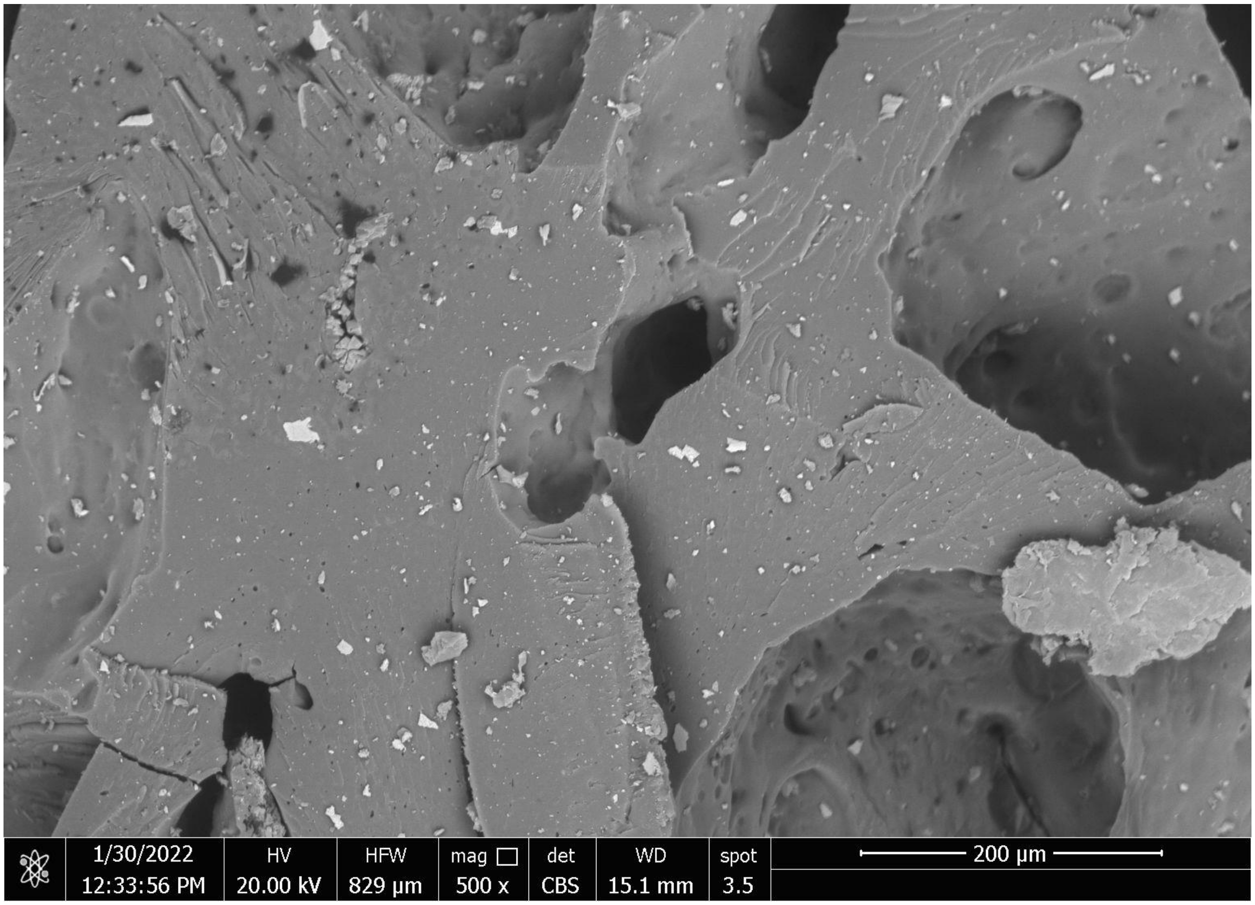

SEM image of Cs-g-4VP/$CuCl_2$

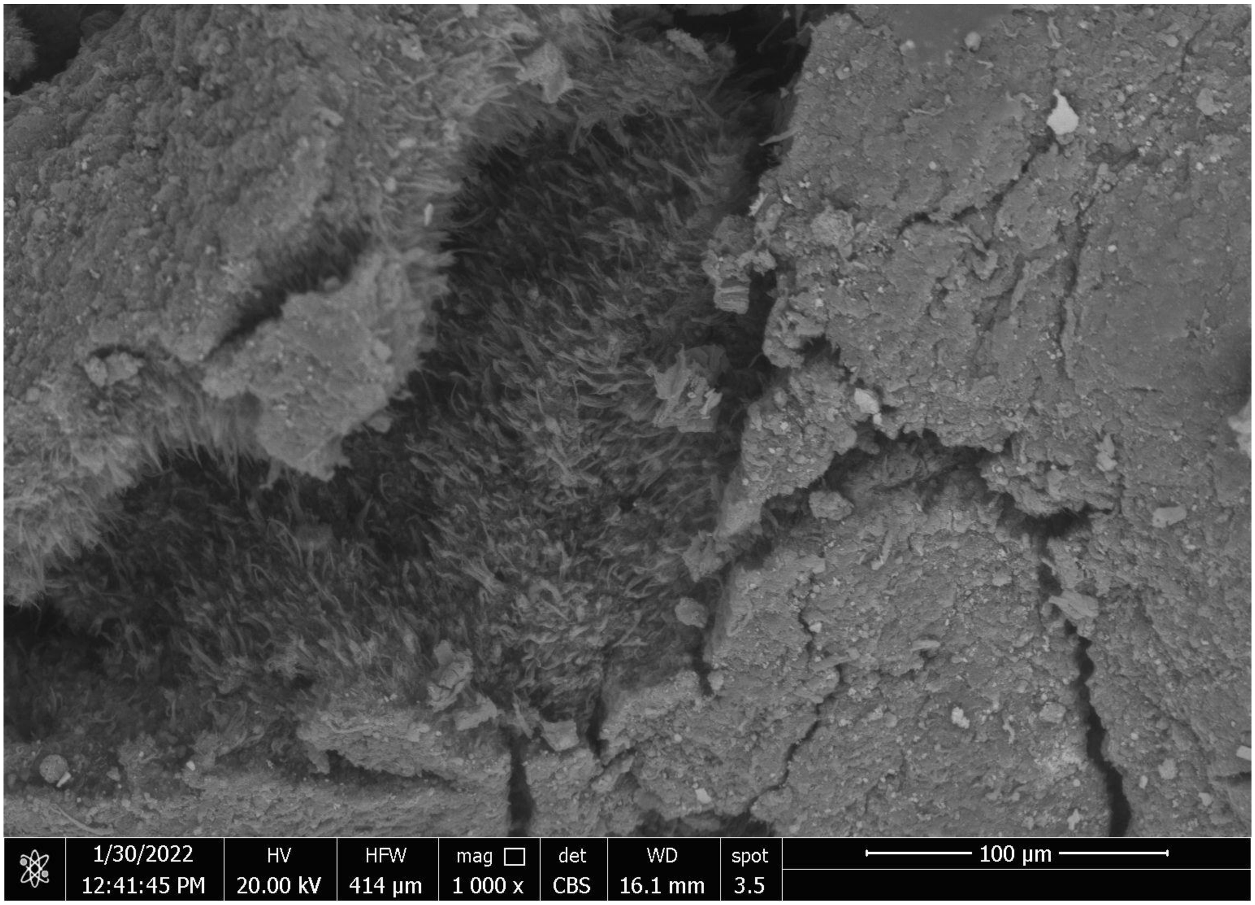

SEM image of Cs-g-4VP/MWCNTs (5% by weight)

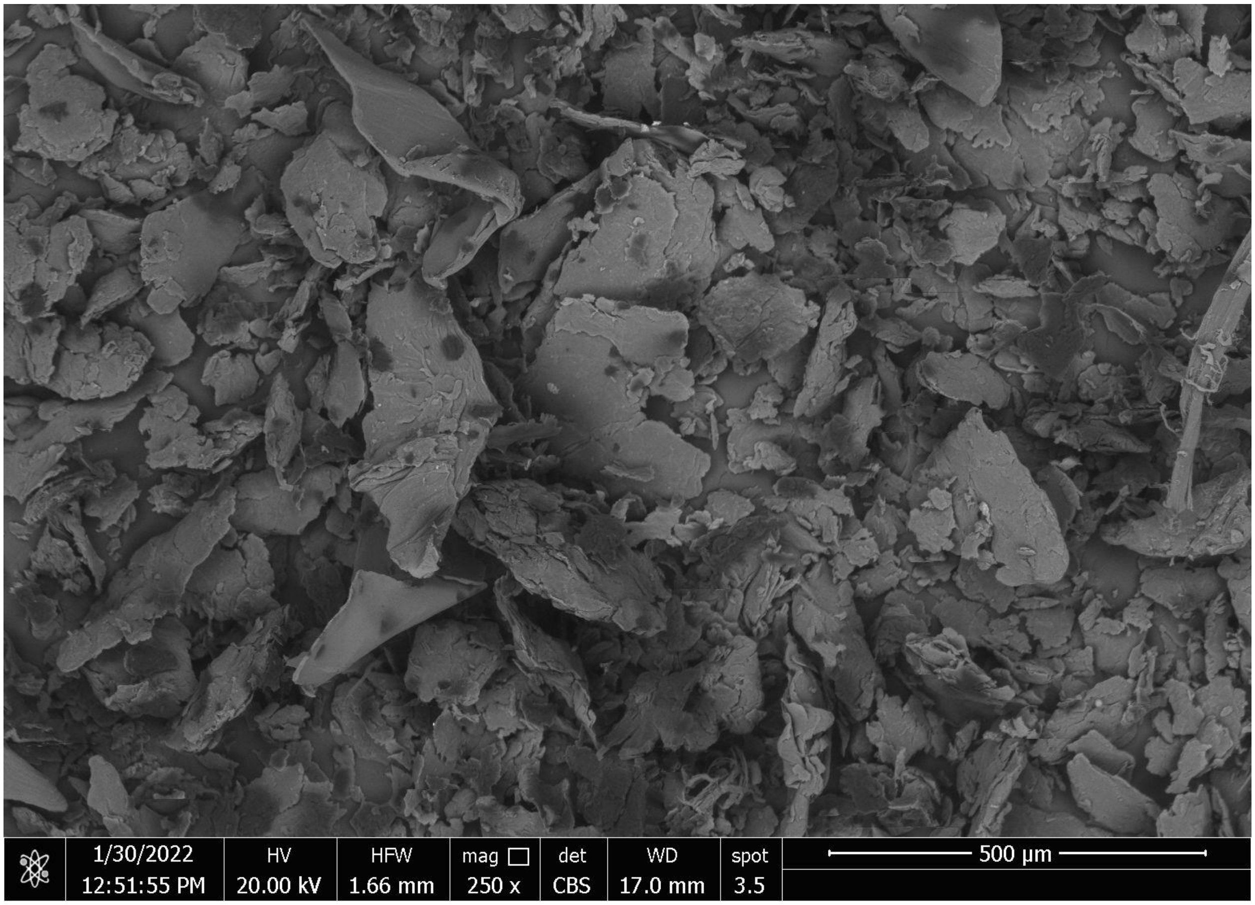

SEM image of Cs

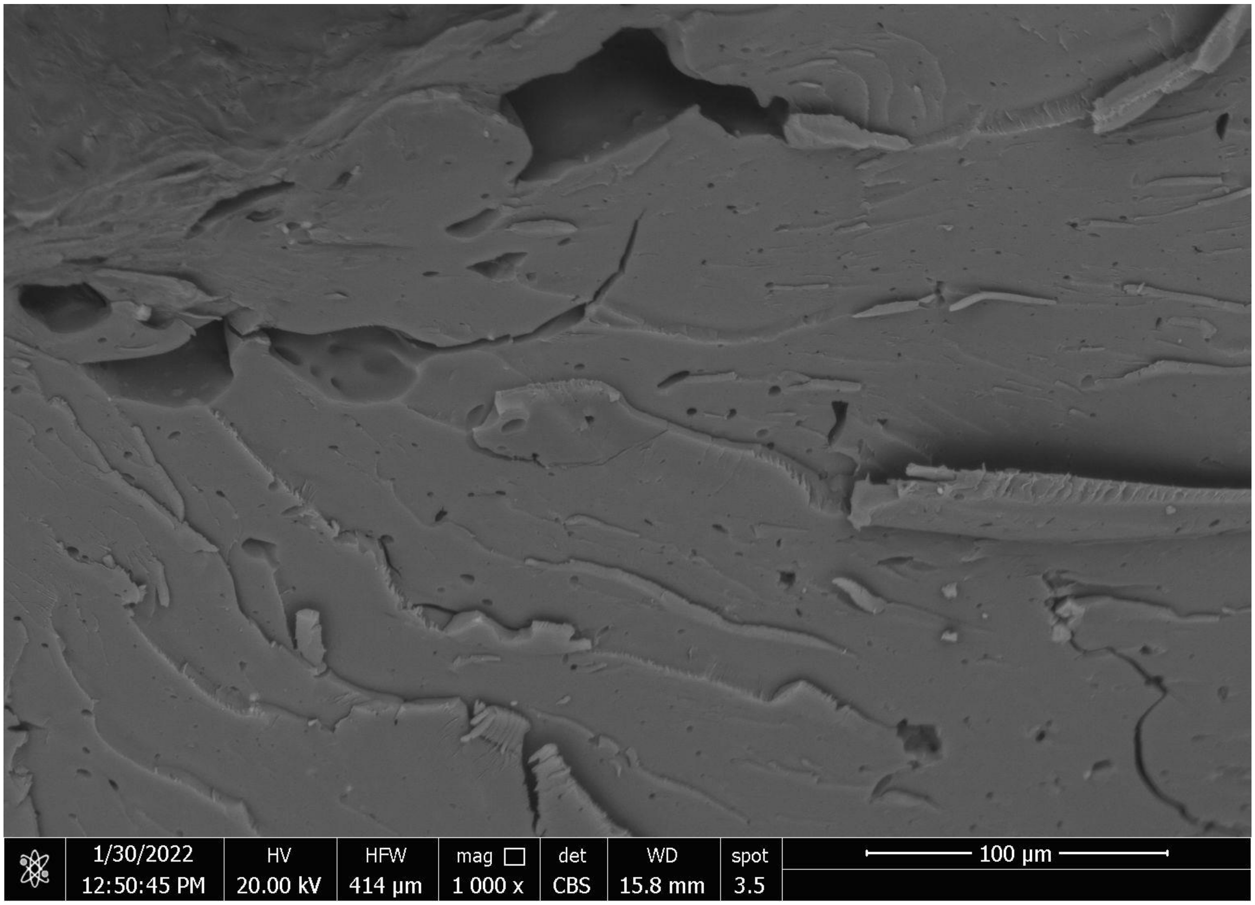

SEM image of 4-VP

Cu(II) ions removal

Sample	Concentration (mg/L)			Cu %	R%
1	740	C0			
2	456			61.62	38.38
3	546			73.78	26.22
4	484			65.41	34.59
5	584			78.92	21.08
6	502			67.83	32.17
7	477			64.46	35.54
8	516			69.73	30.27
9	449			60.68	39.32
10	80	√√	Good	10.81	89.19
11	8.87	√√√	Excellent	1.2	98.8
12	7.33	√√√√	Excellent	0.99	99.01
13	115.8	√	Good	15.64	84.36
14	174.8	√	Good	23.62	76.38

Cu(II) ions removal % as a function of pH and adsorbent masses.

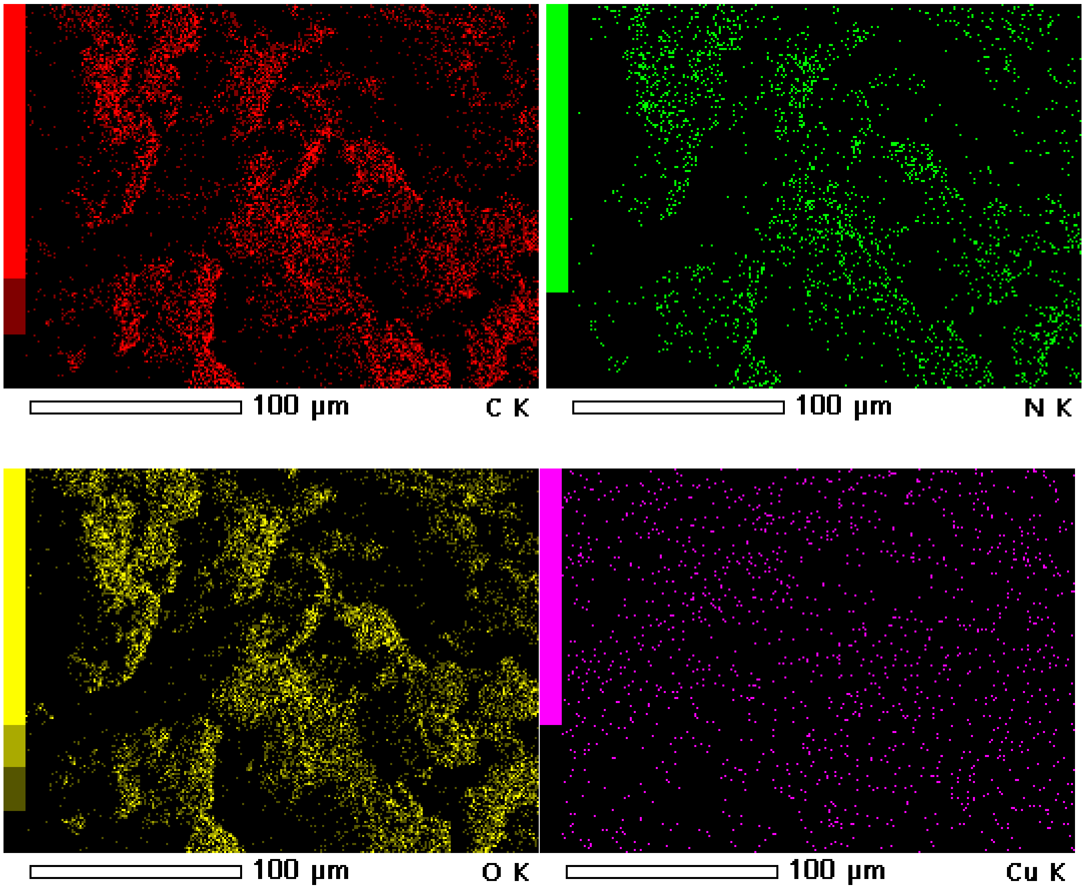

EDX mapping analysis of elemental constitutes of Cs-g-PVC/f-MWCNTs/$CuCl_2$ hybrid
